# Evaluation of efalizumab using safe psoriasis control

**DOI:** 10.1186/1471-5945-6-8

**Published:** 2006-09-19

**Authors:** Kim A Papp, Eric Henninger

**Affiliations:** 1 Probity Medical Research, 135 Union Street East, Waterloo, Ontario, Canada, N2J 1C4, USA; 2 Serono International S.A., 15bis, chemin des Mines, Case postale 54, CH-1211 Geneva 20, Switzerland

## Abstract

**Background:**

Safe Psoriasis Control (SPC) is an important comprehensive measure that is validated for the assessment of benefit:risk of psoriasis treatments, combining efficacy, quality of life, and safety measures. The objective of this analysis was to assess the benefit:risk of efalizumab, a novel biologic agent indicated for the treatment of moderate-to-severe plaque psoriasis, by applying the SPC to data from randomized, placebo-controlled clinical studies of efalizumab.

**Methods:**

SPC was applied to week 12 data from four placebo-controlled, Phase III studies: three retrospective and one prospective, the latter including a cohort of "high-need" patients for whom existing therapies were inadequate or unsuitable.

**Results:**

In the retrospective analysis, 39.4% of patients achieved SPC after 12 weeks of treatment with efalizumab, compared with 10.4% for placebo. In the prospective analysis, 34.3% of patients achieved SPC after 12 weeks of treatment with efalizumab, compared with 7.3% on placebo. Among high-need patients, 33.0% achieved SPC, compared with 3.4% on placebo.

**Conclusion:**

Efalizumab has a favorable benefit:risk profile using the comprehensive outcome measure SPC.

## Background

Over the past decade, technological advancements have resulted in a substantial increase in the number of new molecules under investigation for the treatment of psoriasis. At the same time has come the realization that current efficacy measures have significant limitations in assessing the full therapeutic benefit of psoriasis therapies. Despite its shortcomings, the most widely used measure to assess efficacy of new therapies has remained the Psoriasis Area and Severity Index (PASI) [1,2]. However, concerns regarding this endpoint [3] and the lack of use of PASI in current practice by dermatologists have resulted in the development of alternative measures such as the Salford Psoriasis Index (SPI) [4], the Self Administered PASI (SAPASI) [5], the Koo-Menter Psoriasis Instrument [6], and the National Psoriasis Foundation Psoriasis Score (NPF-PS) [7]**.**

To more fully evaluate and interpret the benefit:risk of new therapies for psoriasis requires measures that assess multiple dimensions of the disease in a clinically meaningful way for patients and physicians alike. Safe Psoriasis Control (SPC) [3] is such a measure that is designed to improve relevance and comparability between studies and drugs by focusing on all clinically relevant outcomes so that accurate assessments can be made regarding new therapies for psoriasis [8].

SPC is an important advance in the evaluation of treatments for psoriasis, and is a useful tool for evaluating the short term benefit:risk profile of new drugs[3]. It is the only assessment that takes into consideration the multiple aspects of psoriasis and its treatment, simultaneously recognizing and incorporating the importance of physician-based assessments of disease severity; patient-based assessments of the impact of the disease; and the safety of the drug.

The data used to derive and validate SPC were for the new biological agent efalizumab, a humanized, recombinant, monoclonal IgG_1 _antibody developed to target multiple T-cell-mediated processes critical to the pathogenesis of psoriasis [9]. More than 3,500 patients have received efalizumab as part of clinical trials for over 3 years, and the data from approximately 2,500 of these were used in the derivation of SPC.

The methodology to derive and validate SPC is discussed in Papp et al [3]. Briefly, important components in the assessment of therapeutic benefit were identified, followed by an analysis to identify clinically meaningful achievements in these components using a statistically robust approach. Thereafter, the endpoint was further validated using a combination of other statistical methodologies, external references, and prospectively in an independent on-going live clinical trial setting.

The purpose of this analysis was to assess the benefit:risk of efalizumab at 12 weeks by applying the SPC to data from randomized, placebo-controlled clinical studies of efalizumab.

## Methods

As outlined in Papp et al [3], three levels of SPC are described in order to demonstrate the accuracy, validity and rigor of the multidimensional measure. Dimensions are efficacy (assessed using PASI data), safety (assessed using adverse events data), and quality of life, which is assessed using data from the Dermatology Quality Life Index (DLQI), a reliable and validated self-administered dermatology quality of life instrument [10]. For this paper, however, in recognition of the practical necessities of normal clinical practice, only one was selected. Given that a DLQI score of 6 is considered to represent a normal quality of life [11], it was decided for practical clinical purposes to use good SPC (defined as PASI score ≤8 and DLQI score ≤6 with no SAEs and no severe AEs related to study drug and not withdrawn) as the target endpoint.

In this paper, the good SPC endpoint was applied to efalizumab clinical trial data as described in the following section (Table 1).

### Retrospective application

SPC was applied retrospectively to week 12 data from studies that had been completed prior to derivation of the endpoint. In this regard, data analyses were performed on the intention-to-treat (ITT) subject populations enrolled into three North American randomized, placebo-controlled, Phase III clinical trials of efalizumab (ACD 2058, ACD 2059, and ACD 2390) [12-15]. Data from each study were analyzed on an individual as well as pooled basis. The first 12 weeks of all phase III trials were double-blind, parallel group design. Efalizumab was given subcutaneously at 1 mg/kg/week.

### Prospective application

SPC was applied prospectively to week 12 data from studies that were completed after derivation of the endpoint. For this paper data analyses were performed on the ITT subject populations enrolled in an international randomized, double-blind, placebo-controlled, parallel-group, multicentre trial (IMP 24011, ClinicalTrial.gov registration number NCT00256139) consisting of an initial 12-week, double-blind treatment period. PASI 75 responders entered an observation period until relapse or up to 24 weeks, after which patients received 12 weeks of open-label re-treatment. Non-responders went directly to an extended treatment period of 12 weeks. The primary objective of the study was to evaluate the safety and efficacy of efalizumab 1.0 mg kg^-1 ^given subcutaneously once weekly for 12 weeks compared with placebo in moderate-to-severe patients, and in a cohort of "high-need" patients for whom existing therapies were inadequate or unsuitable. An initial cohort of moderate-to-severe patients was recruited, on whom an interim analysis was performed showing efalizumab efficacy versus placebo. After this, only high-need patients were recruited.

High-need patients were defined as those patients who had failed treatment on, were intolerant of, or had contraindications to at least two currently available systemic therapies (e.g., photochemotherapy, cyclosporin, corticosteroids, methotrexate, oral retinoids, mycophenolate mofetil, thioguanine, hydroxyurea, sirolimus, azathioprine, 6-mercaptopurine).

SPC was analyzed for the first 12-week treatment period for which data are available.

### Patients

Inclusion and exclusion criteria were similar for all studies included in the analysis, except for the high-need cohort, as already explained. Patients were required either to have received previous systemic treatment for psoriasis or to be a candidate for such treatment without prior history. All patients enrolled were 18 to 75 years old with at least a 6-month history of moderate-to-severe plaque psoriasis covering ≥10% of total body surface area (BSA), with a minimum PASI of 12.0 at screening.

The total number of all patients for the prospective analysis using study IMP 24011 was 793, of whom 526 were high-need patients.

### Treatment

Study treatment efalizumab 1 mg/kg/week was administered via subcutaneous injection once weekly for 12 weeks. The first weekly dose of efalizumab was given at a conditioning dose of 0.7 mg kg^-1^, followed by the full dose level of 1.0 mg kg^-1^. No other systemic psoriasis therapy or phototherapy was allowed during the trial.

### Assessment of benefit:risk

Conventional physician-assessed endpoint data such as PASI, and patient outcome measures such as DLQI, were collected for the 12-week timepoint. Safety was assessed in terms of the incidence, severity, and relationship to treatment of adverse events (including serious adverse events) and the incidence of treatment-emergent laboratory abnormalities. SPC for efalizumab was calculated from these 12-week data.

## Results

### Retrospective SPC analysis

Table 2 shows the SPC outcome data from the retrospective analyses. The results were similar between the studies. At 12 weeks, SPC was achieved by 46.3%, 37.1%, and 37.9% of efalizumab-treated patients for studies ACD 2058, ACD 2059, and ACD 2390, respectively. When pooled, 39.4% of patients achieved SPC at week 12, compared with 10.4% for placebo.

### Prospective SPC analysis in study IMP 24011

In the study IMP 24011, DLQI was administrated to 494 of the 793 patients (165 randomized to placebo and 329 to efalizumab 1.0 mg/kg/wk), as appropriately validated translations of the questionnaire were not available for use in Greece, Israel, Portugal or Russia. Thus, determination of the percentages of patients achieving SPC at week 12 was based on the cohort of patients for whom DLQI was available. In these patients 34.3% of patients achieved SPC on efalizumab, compared with 7.3% on placebo (Table 3).

**Figure 1:**
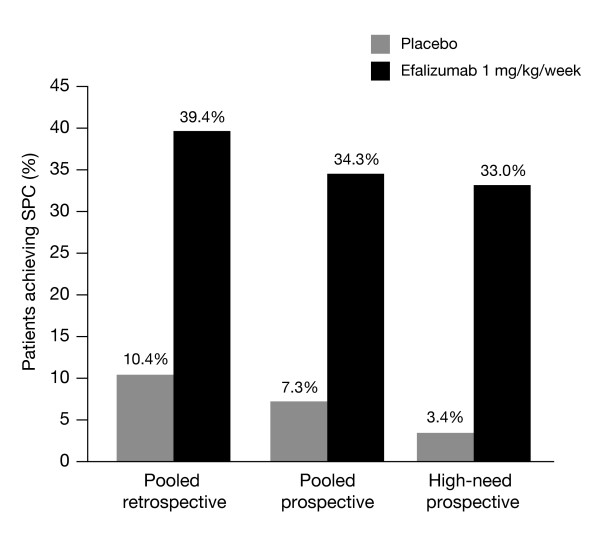
Patients achieving SPC on efalizumab at week 12.

In high-need patients, only 4/117 patients (3.4%) given placebo achieved SPC at week 12, compared with 73/221 (33.0%) on efalizumab (Figure 1).

Observed response rates at week 12 were lower in the prospective validation compared with the retrospective rates. However, the rate differences between active and placebo observed in the prospective study (27% for the total study group and 30% for the high need group) were similar to that seen in the retrospective analyses (29%).

### Comparison of PASI-75 and SPC

**Figure 2:**
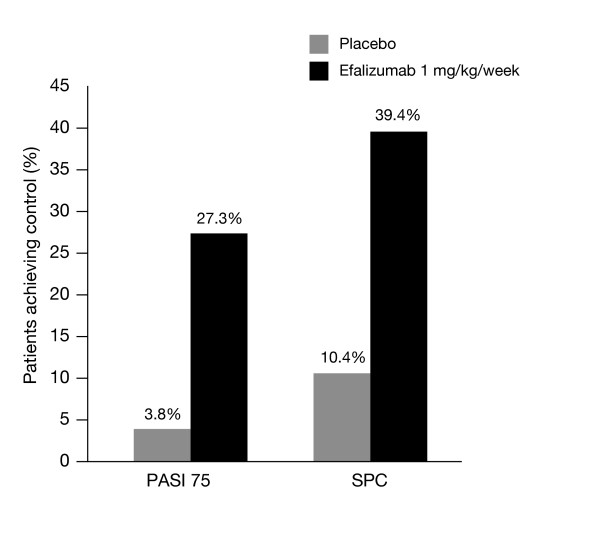
Comparison of PASI 75 and SPC for pooled retrospective data at week 12.

The efficacy-only measure, PASI 75, was compared with the multidimensional SPC for pooled retrospective data from the three studies (ACD 2058, 2059, and 2390). This showed that 27.3% of patients treated with efalizumab achieved PASI 75 at week 12, while 39.4% of patients achieved SPC (Figure 2).

## Discussion and conclusion

Efalizumab has been studied in more than 3500 patients. This extensive database offers an ideal source of information for assessing traditional measures of efficacy, and generating new measures of benefit to assess whether a patient's psoriasis is controlled. The SPC assessment moves away from an approach focused primarily on treatment difference in PASI 75 response in isolation, to an approach that would further characterize each patient's response to treatment, leading to a better understanding of the benefit:risk that should be expected in psoriasis treatment.

Using conventional, uni-dimensional assessment measures such as PASI 75 and DLQI, efalizumab has already been demonstrated to be significantly more effective than placebo. However, the use of these physician- or patient-assessed scales either alone, or independently of each other, does not accurately demonstrate the multidimensional effect that efalizumab has in safely controlling psoriasis. Studies indicate that there is a need to consider psoriasis and its treatment not just in terms of objective assessments of lesion severity, but also in terms of the impact of the disease on patients' lives [16,17]. The inclusion of the safety component in the SPC endpoint allows a simple benefit:risk assessment to be made, as the endpoint describes the proportion of patients who had benefit without major side effects.

We applied this new outcome measure – SPC – using week 12 data obtained from studies that had already been completed and analyzed using conventional uni-dimensional measures such as PASI, and evaluated SPC prospectively in a new study (IMP 24011) that had not been completed at the time SPC was described.

A favorable overall benefit:risk profile as determined by the SPC endpoint was confirmed in nearly 40% of all the patients treated with efalizumab for 12 weeks. Using SPC in both prospective and retrospective clinical trial analyses, efalizumab consistently demonstrated superiority over placebo, demonstrating that many patients achieve disease control without major safety issues, and at the same time experience an improved quality of life. Importantly, efalizumab was similarly effective in high-need patients. Also, even amongst those patients who did not manage to achieve SPC, approximately 9% achieved PASI 75 after 12 weeks of treatment with efalizumab 1.0 mg/kg, compared with less than 1% of patients on placebo.

Improvement in the management of psoriasis is particularly welcome as the impact of the disease, particularly among high-need patients, and shortcomings of current treatments are becoming more widely recognized. Current management of patients with moderate-to-severe plaque psoriasis involves systemic therapies, such as cyclosporin, methotrexate, acitretin and PUVA, which are associated with treatment-limiting systemic cumulative toxicities. The use of biological agents such as efalizumab that modulate the activation of T-cells and their migration into the dermal and epidermal tissues may provide more targeted therapy with an improved safety profile. Our findings, and those of prior trials of efalizumab, support this concept and demonstrate promising opportunities to improve therapy. The observations of substantial disease improvements with little evidence of systemic toxicity are consistent with those in previous reports.

The endpoints used in trials should align with the treatment goals of clinical practice. SPC includes all relevant assessments (physician, patient, and safety) and is a more robust way of estimating the true benefit:risk ratio of psoriasis treatment. The findings of this analysis using the SPC show that efalizumab provides significant improvements safely in patients with moderate-to-severe plaque psoriasis, and particularly in difficult-to-treat, high-need patients.

## Competing interests

Kim Papp has acted as a consultant to Serono

Eric Henninger is an employee of Serono

*** This material was presented as a poster during the EADV 2004 convention

## Authors' contributions

Both KP and EH developed and applied the Safe Psoriasis Control approach to efalizumab clinical trial data. KP was an investigator in several efalizumab trials. Both authors contributed to the preparation of this manuscript.

## Pre-publication history

The pre-publication history for this paper can be accessed here:



## Figures and Tables

**Table 1:** Studies used for retrospective and prospective application of SPC

**Study Number**	**Study Title**	**Number of Patients**	**Application analysis of Good Safe Psoriasis Control (SPC)**
ACD 2058[14]	"A Phase III, Randomized, Double-Blind, Parallel-Group, Placebo-Controlled, Multicenter, Multidose Study to Evaluate the Efficacy and Safety of Subcutaneously Administered Anti-CD11a in Adults with Moderate to Severe Plaque Psoriasis"	498 subjects •efalizumab 1 mg/kg – 162 •efalizumab 2 mg/kg – 166 •placebo – 170	Retrospective
ACD 2059[13]	"A Phase III, Randomized, Double-Blind, Parallel-Group, Placebo-Controlled, Multicenter, Multidose Study to Evaluate the Efficacy and Safety of Subcutaneously Administered Anti-CD11a in Adults with Moderate to Severe Plaque Psoriasis Who Are Candidates for Systemic Therapy"	597 subjects •efalizumab 1 mg/kg – 232 •efalizumab 2 mg/kg – 243 •placebo – 122	Retrospective
ACD 2390[12]	"A Phase IIIb, Randomized, Double-Blind, Parallel-Group, Placebo-Controlled, Multicenter Study to Evaluate the Efficacy and Safety of 1.0 mg/kg Subcutaneously Administered Efalizumab in Adults with Moderate to Severe Plaque Psoriasis"	556 subjects •efalizumab 1 mg/kg – 369 •placebo – 187	Retrospective
IMP 24011[15]	"A multicentre, randomised, double blind, placebo controlled phase III study of subcutaneously administered Raptiva in the treatment of patients with moderate to severe psoriasis"	793 subjects •efalizumab 1 mg/kg – 529 •placebo – 264 •(526 high-need)	Prospective

**Table 2:** Retrospective SPC results (Good control level) at week 12

Study	Treatment Group	Safe Psoriasis Control (SPC)*
ACD2058[14]	Placebo (n = 170)	18 (10.6 %)
	Efalizumab 1.0 mg/kg/wk (n = 162)	75 (46.3 %)
ACD2059[13]	Placebo (n = 122)	12 (9.8 %)
	Efalizumab 1.0 mg/kg/wk (n = 232)	86 (37.1 %)
ACD2390[12]	Placebo (n = 187)	20 (10.7 %)
	Efalizumab 1.0 mg/kg/wk (n = 369)	140 (37.9 %)
Pooled studies	Placebo (n = 479)	50 (10.4%)
	Efalizumab 1.0 mg/kg/wk (n = 763)	301 (39.4%)

* SPC is defined by PASI <= 8 and DLQI <= 6 and No SAEs and No Severe AEs related to Study Drug and not withdrawn

**Table 3:** Prospective SPC results at week 12

Study IMP 24011[15]	Treatment Group	Safe Psoriasis Control (SPC)*
Total study group	Placebo (n = 165)	12 (7.3 %)
	Efalizumab 1.0 mg/kg/wk (n = 329)	113 (34.3 %)
High need group	Placebo (n = 117)	4 (3.4 %)
	Efalizumab 1.0 mg/kg/wk (n = 221)	73 (33.0 %)

* SPC is defined by PASI <= 8 and DLQI <= 6 and No SAEs and No Severe AEs related to Study Drug and not withdrawn
